# Fabrication of Free-Standing Gelatin Thin Films via the Gelation and Drying of Liquid Foam Films

**DOI:** 10.1021/acs.langmuir.5c03893

**Published:** 2025-10-14

**Authors:** Ashesh Garai, Sadaki Samitsu, Miwa Ohniwa, Izumi Ichinose

**Affiliations:** † 660446 Rammohan College, 102/1 Raja Rammohan Sarani, Kolkata 700009, India; ‡ Research Center for Macromolecules and Biomaterials, 52747 National Institute for Materials Science, 1-2-1 Sengen, Tsukuba, Ibaraki 305-0047, Japan

## Abstract

Ultrathin foam films can be prepared from numerous compounds under a wide range of conditions but have a narrow application scope because of their small size, susceptibility to rupture, and other drawbacks arising from low mechanical stability of the bilayer structure. To address this gap, we herein prepared ultrathin foam films of gelatin using a facile method via drying thin liquid films of aqueous gelatin solutions and the gelation. Their thickness ranges between nanometers to micrometers in response to changes in process parameters. The effects of various process parameters, including the solution concentration, frame size, drying temperature, and humidity, were systematically investigated. In addition to surface tension affecting the initial formation of the liquid film, the high viscosity of the solutions stabilized the film and caused gelation. The key formation factor of the dried foam films was explained by the sol–gel behavior of aqueous gelatin solutions. The prepared films are mechanically more stable than surfactant films and therefore have a wide range of potential applications as separation membranes. Glutaraldehyde cross-linking makes these films water-insoluble and therefore suitable for use as a nanoseparation membrane. Fully cross-linked gelatin thin films showed a rejection performance of 100% for 5 and 2 nm gold colloids, Direct Yellow 1, and potassium ferricyanide.

## Graphical abstract

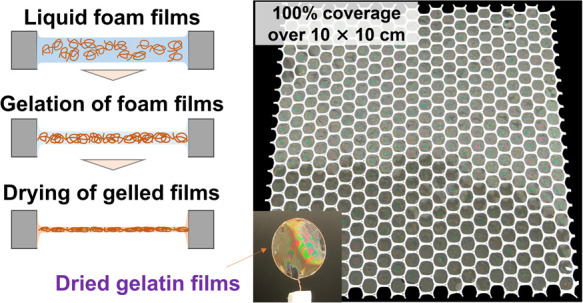

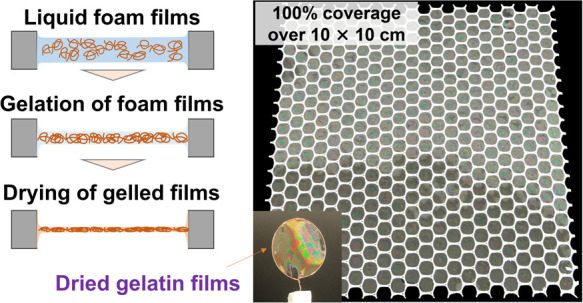

## Introduction

Aqueous solutions containing surfactants can form thin liquid films supported by solid frames, similar to soap films. When the liquid-film thickness decreases to less-than-submicron values because of drainage, interference colors are observed. Upon further thinning to <10 nm, the interference colors disappear, and the film turns black.^1,2^ Thin liquid films sandwiched between two air–water interfaces and stabilized by surface-active species are sometimes referred to as liquid foam films.^3^ Liquid foam films have long been studied as a basic model of foams and emulsions used in healthcare, household products, and food applications.^3–5^ The stability of liquid foam films is governed by drainage dynamics and influenced by capillary phenomena, hydrodynamics, interfacial transport and rheology, and intermolecular interactions.^6,7^ Vermant et al.^7^ analytically formulated the dynamics of liquid foam films based on a continuum model. Although the stability of a liquid foam film at equilibrium can be evaluated using a concise formula describing the disjoining pressure, the molecular origins of the corresponding physical parameters are rather complex and influenced by not only process parameters (e.g., temperature, concentration, pH, and interfacial tension) but also molecular parameters (e.g., surfactant hydrophilicity, ionic species, steric repulsion, and type of water-holding additives (alcohols and sugars with multiple hydroxyl groups).^8–10^

In a closed space with a constant temperature, pressure, and relative humidity (RH), a liquid foam film exists in an equilibrium state and has a long lifetime. When held in an open space, most liquid foam films become unstable and prone to rupture under nonequilibrium conditions due to liquid evaporation, temperature changes, fluctuations in environmental pressure, etc.,^11–13^ solidifying into thin dried films without rupturing only in the presence of optimal surfactants and under optimal process conditions.

Freestanding thin films mechanically stable under atmospheric conditions have a broad range of potential applications, such as separation membranes and high-sensitivity sensors.^14,15^ Based on this principle, Ichinose et al. reported the demonstration of ultrathin dried foam films composed of low-molecular-weight surfactants with long alkyl chains and amphiphilic ionic liquids.^16–20^ For example, when a dilute aqueous solution of dodecyltrimethylammonium bromide was held in the aperture of a 7 μm square copper grid, a dried foam film with a thickness of several nanometers was obtained. High-coverage ultrathin films were also obtained using double-chain (dodecyldimethylammonium bromide) and zwitterionic (dodecylphosphocholine) surfactants.^16^ Owing to their stability in high vacuum and up to 100–150 °C, these films could be coated with a variety of metals, inorganics, and carbons by thermal evaporation or ion sputtering.^18^ However, such freestanding ultrathin films of low-molecular-weight surfactants are difficult to handle because of their insufficient mechanical strength and tendency to rupture, and the size of these films is currently limited to less than several tens of micrometers.^16^

In addition to low-molecular-weight surfactants, large surface-active species such as amphiphilic and hydrophilic polymers, proteins, and micro- and nanoparticles can also stabilize liquid foam films, although the corresponding complex formation mechanisms are being intensively investigated.^21,22^ Such macromolecular surfactants can form large-area freestanding dried foam films that have a sufficient mechanical strength, are transferable, and can be functionalized through surface coating. Brij-35, a poly­(ethylene oxide)–based surfactant with a molecular weight of ∼ 1200 Da afforded a stable 200 nm–thick film with a diameter of 2 cm.^19^ Chen et al. fabricated centimeter-sized dried foam films via the evaporation-induced self-assembly of graphene oxide nanosheets driven by π-π stacking interactions and hydrogen bonds between adjacent nanosheets.^23^ Andrieux et al. investigated freestanding thin foam films of alginate hydrogels using a home-built microfluidic thin-film pressure balance system and elucidated the effects of gelation and drying on pore-opening and rupture behavior.^24^

Surface-active nanostructured proteins are useful bio/functional materials for the targeted delivery^25^ and controlled release of drugs,^26,27^ tissue engineering,^28,29^ wet adhesion,^30^ coating layers for nanofabrication,^31^ and separation membranes.^32,33^ Protein thin films have been prepared by various methods, including layer-by-layer deposition,^34^ spin coating,^35,36^ laser deposition,^37^ and filtration.^32,33^ However, facile methods of fabricating large-area free-standing protein thin films are challenging. Owing to the unique molecular structures and multifunctional chemistry of proteins, such films hold promise as separation membranes for the direct capture of carbon dioxide from ambient air.^38–40^ Among the various proteins, gelatin exhibits the advantages of excellent film mechanical properties, gelation behavior upon heating and cooling, and industrial availability.^41,42^

Herein, we focus on the surface activity and thermal gelation behavior of proteins and demonstrate that large-area freestanding thin films stable even in the dry state can be produced from gelatin solutions at an optimal temperature, concentration, viscosity, RH, and surface tension. Moreover, we investigate the correlation between the dry film–forming capability and sol–gel behavior of gelation solutions, solution properties (e.g., viscosity and surface tension), and drying conditions. The obtained films can be transferred to solid substrates by simple attachment to the same and cross-linked using common protein cross-linking agents to impart stability against hot water. In a practical utility demonstration, the obtained thin films are used as separation membranes for removing gold nanocolloids and dye molecules from water.

## Experimental Section

### Materials

Type A gelatin derived from porcine skin (gel strength ∼ 300 g Bloom) and lyophilized albumin powder derived from chicken egg white were purchased from Sigma-Aldrich. Aqueous glutaraldehyde (50 wt %) was purchased from Tokyo Chemical Industry Co., Ltd. Genipin and reagent-grade 99.5% ethanol were purchased from Wako Pure Chemical Industries, Ltd. Milli-Q water with a resistivity of 18.2 MΩ·cm at 25 °C was used.

### Preparation of Gelatin Films

Gelatin was dissolved in water at 45–60 °C over 1 h upon magnetic stirring. The resulting hot solution (0.5–5 wt %) was passed through a syringe filter with a 0.2 μm hydrophilic polytetrafluoroethylene membrane, and the filtrate was sonicated at 100 W for 15 min to remove air bubbles and kept at a specific temperature (45–60 °C) for thin film preparation. Freshly prepared solutions were preferred; when storage was needed, the solutions were kept in a refrigerator and reheated before use. The ability to form liquid foam films markedly deteriorated after 4 days of storage at 45 °C. Gelatin films were prepared by immersing a handmade circular copper frame (thickness of copper wire = 0.28 mm, diameter of copper frame = 1.0–14.6 cm, cleaned by immersing in acetone overnight) into the above filtrates, carefully lifting the frame, and air-drying the foam film in a horizontal orientation without agitation. Drying was performed for 15–30 min, with the exact drying time depending on the drying temperature (10–55 °C) and RH (10–70%). Drying temperature and humidity were controlled using a benchtop type temperature humidity chamber (SH-242, ESPEC Corp., Japan). Ambient conditions corresponded to 24 ± 2 °C and 40 ± 20% RH (Scheme 1).

**Scheme 1:**
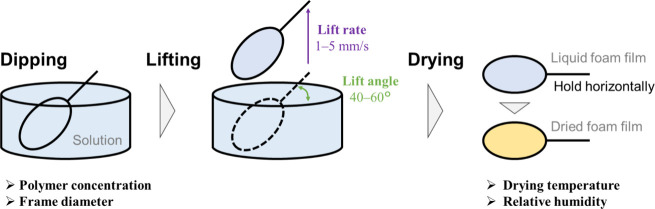
Fabrication Procedure of Dried Foam Films

Liquid foam films were easily prepared by holding the frame by hand, dipping it into the aqueous gelatin solution, and lifting it out. To ensure reproducible film formation, the lifting angle (0–90°) and lifting speed (0.1–10 mm/s) were optimized using a microdip instrument (model MD-0408). Large-area films were prepared using polypropylene (4 mm arm), polyester (5 mm arm), and nylon (2 cm arm) meshes. The films were cross-linked with glutaraldehyde and genipin. A solution of glutaraldehyde was prepared by diluting aqueous 50 wt % glutaraldehyde with ethanol. Genipin-cross-linked films were prepared using a solution containing both gelatin and genipin, which was prepared by dissolving gelatin (200 mg) in water (9.5 mL) at 60 °C with magnetic stirring for 3 h, after which genipin (20 mg) in ethanol (0.5 mL) was added at room temperature. The mixture was aged at 55 °C for 1 h and then used to prepare foam films. The originally colorless genipin solution became purple and then blue during aging. The cross-linked gelatin films were taken out from the solution and dried at room temperature for 1 day.

### Characterization

Film morphology and thickness were evaluated using scanning electron microscopy (SEM; S-4800, Hitachi High-Tech Corp.; acceleration voltage = 10 kV, current = 5 μA). To prevent the electric charging of the gelatin films, the films were transferred on a polycarbonate membrane and coated them with a 2 nm–thick platinum layer using an ion sputterer equipped with a quartz crystal microbalance (E-1030, Hitachi, Ltd.; 10 Pa of argon, 10 mA). The surface tensions of gelatin solutions were measured at 30–60 °C according to the Wilhelmy plate method using a Kruss tensiometer, while the corresponding viscosities were measured using an advanced rheometer (AR G2, TA Instruments). A parallel plate with a solvent trap was mounted on top of a Peltier plate temperature controller at a gap width of 500 μm. The Fourier transform infrared (FTIR) spectra of the films were recorded using an FTIR spectrometer (FTIR-8400S, Shimadzu Corp.) in transmission mode. Free-standing films were placed in the optical path so that the infrared beam passed through the film center.

## Results and Discussion

At appropriate concentrations and wire frame diameters, liquid films formed on the wire frame similarly to the liquid foam films of low-molecular-weight surfactants. When the long-lasting and rupture-resistant liquid films were dried under appropriate conditions, dried gelatin films were formed. The dried film obtained using a 1.5 wt % gelatin solution and 1 cm–diameter wire frame showed bright iridescent colors due to optical interference, suggesting a thickness of less than a few μm (Figure 1a). To examine the effect of gelatin concentration, we examined dried films obtained from 5, 3, and 1 wt % gelatin solutions using a 2 cm–diameter frame upon drying at ambient conditions. According to the results of cross-sectional SEM analysis, films prepared from the 5, 3, and 1 wt % solutions had central-part thicknesses of 4.0 ± 0.8 μm (), 1.3 ± 0.4 μm (), and 260 ± 150 nm (Figure 1b), respectively. As expected, film thickness decreased with the decreasing gelatin concentration. The Fourier transform infrared spectra of the above films (Figure 1c) featured the amide II, amide I, amide B, and amide A bands of gelatin at 1552, 1654, 3082, and 3324 cm^–1^, respectively.^38,39^ The absorbance and, hence, film thickness, decreased with the decreasing gelatin concentration, in agreement with the results of SEM analysis.

**Figure 1:**
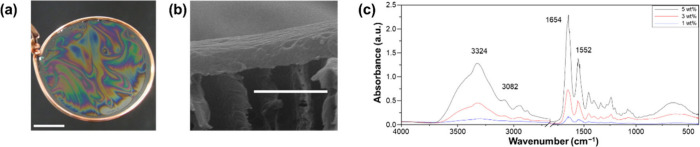
(a) Photograph of a freestanding dried gelatin film produced using a 1.5 wt % gelatin solution, (b) cross-sectional SEM image of a film produced using a 1.0 wt % gelatin solution, and (c) Fourier transform infrared spectra of different gelatin films. Scale bars: (a) 2.5 mm, (b) 1 μm.

We also tested rhombus-shaped polypropylene and hexagonal polyester meshes to prepare freestanding dried gelatin films from gelatin solutions of various concentrations at 55 °C and a relative humidity of 25–30%. The coverage percentage depended on the gelatin concentration, solution temperature, and humidity. Films with 100% coverage were obtained from a 2 wt % gelatin solution at ambient conditions using polypropylene and polyester meshes (Figures 2a and 2b, respectively). The dried film thickness was not uniform, increasing upon moving from the center to the frame (Figure 2c). The film produced using a polymer mesh with 4 mm openings and 3 wt % gelatin solution featured a thickness of 0.7 μm at the center, which was 2.4 times smaller than that near the frame. When a 1.5 wt % gelatin solution was used, the film thickness was 100 nm in the center and 400 nm near the frame. This difference in thickness between the center and near-frame regions originated from the drying process. As the liquid film was held horizontally, the surface tension of the liquid caused the excess solution in the liquid film to be drawn toward the frame. The film thickness near the frame became greater than that at the center, which allowed the dried foam film to be stably fixed to the frame and resulted in a large size and coverage area. Figure 2e shows a photograph of a dried foam film prepared using a 5 wt % gelatin solution and 14.6 cm–diameter wire frame. The center of the film was thinner than its edges, and iridescent colors appeared because of optical interference. The central part (1.7 ± 0.8 μm thick) was thinner than the film prepared from a 5 wt % solution (4.0 ± 0.8 μm thick, ). The thickness of the central part depended not only on the gelatin concentration but also on the frame diameter, decreasing with the increasing frame size. Figure 2d presents the coverage percentage averaged over a 10 cm × 10 cm region as a function of the gelatin concentration. For both polypropylene and polyester meshes, the coverage was almost 100% at concentrations of ≥ 1.5 wt % but substantially decreased at lower concentrations, with almost no coverage observed at ≤ 0.5 wt %. Thus, the gelatin concentration was positively correlated with the film thickness and coverage, which resulted in a trade-off between the demands for low thickness and high coverage in practical applications. When a 4 mm polypropylene mesh was applied to a 1 wt % gelatin solution, Newton black films formed in the central parts of the dried gelatin film (Figure 2f); however, this film could not cover all mesh openings (Figure 2d).

**Figure 2:**
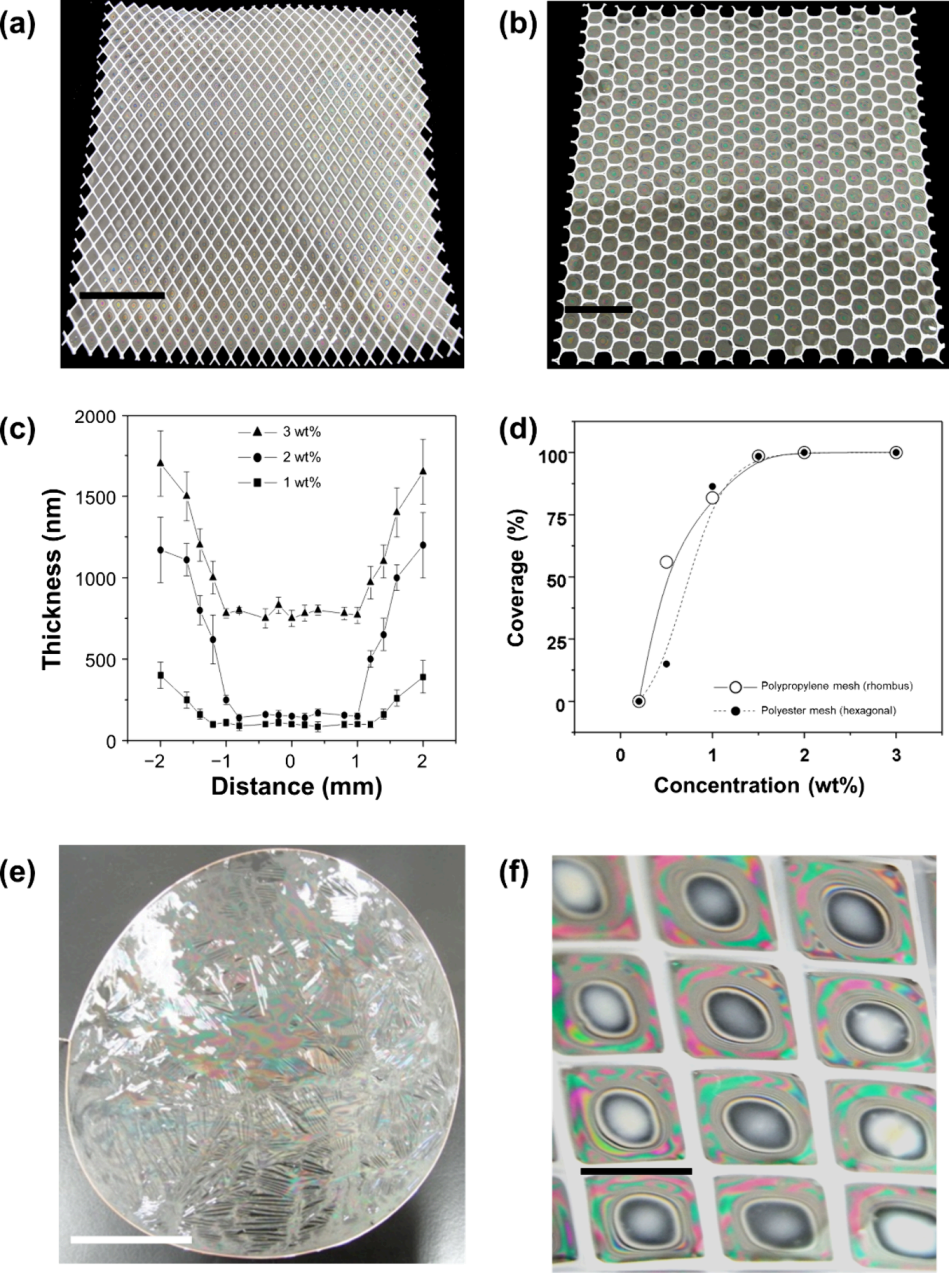
Photographs of dried gelatin films over a 10 cm × 10 cm area with 100% coverage prepared from a 2.0 wt % gelatin solution using (a) 4 mm polypropylene and (b) 5 mm polyester meshes. (c) Thickness profiles of dried films obtained using different gelatin concentrations and a polypropylene mesh (zero denotes the center, negative/positive distances denote regions to the left/right from the center, respectively). (d) Coverage percentages of films prepared using different gelatin concentrations and polymer meshes. (e) Photograph of a large-area dried gelatin film obtained using a 5.0 wt % gelatin solution and 14.6 cm–diameter frame. (f) Newton black films in the central parts of a dried gelatin film prepared using a polypropylene mesh with 4 mm openings and 1 wt % gelatin solution. Scale bars: (a, b) 2 cm, (e) 4 cm, (f) 4 mm.

The weight of the liquid film was recorded using an electronic balance while the frame was kept horizontal after removal from the gelatin solution (). Assuming a solution density of 1 g/cm^3^ and uniform thickness, the film thickness was approximated by dividing the film weight by the frame opening area. A 1 cm–diameter frame removed from a 2 wt % solution formed a liquid film with an average thickness of 92 μm, whereas a 3 cm–diameter frame removed from a 4 wt % solution formed a 39 μm–thick liquid film. Despite the higher solution concentration in the latter case, the corresponding liquid film was thinner, in agreement with the decrease in the dry-film thickness at the center with the increasing frame size. The time-dependent change in the liquid-film weight was measured under ambient conditions (24 ± 2 °C, 35–45% RH). Regardless of the liquid-film size, the weight decreased with time and was below the measurement limit (<0.1 mg) after 15–25 min (), which indicated the completion of water evaporation. Under appropriate drying conditions, the gelatin film remained in the frame without rupturing.

The success rate of dry-film formation depended on the lifting angle and lifting speed, with the optimal conditions determined as lifting angle = 45–60° and lifting speed = 1–5 mm s^–1^ (). We also examined the effects of other parameters related to dry-film formation, namely the gelatin concentration, temperature and RH during drying, and wire frame diameter. Five 1 cm-diameter frames were immersed into 2 and 4 wt % solutions, and the formation of dried films was investigated under various drying conditions in the temperature range of 10–55 °C and RH range of 10–70% (Figures 3a and 3b). When dried at 40 or 55 °C and 70% RH, all five films ruptured within several minutes after the onset of drying, and no dried films formed. Conversely, at 15 °C and 40% or 70% RH, all liquid films dried without rupturing, and dried foam films were obtained with good reproducibility. The upper-limit temperature and RH values at which dried films could be formed increased with increasing solution concentration. Dried films were formed reproducibly at ≤ 25 and ≤ 30 °C for 2 and 4 wt % solutions, respectively. Interestingly, at 70% RH, a flat-surface film was obtained, whereas a wrinkled-surface film was obtained at 40% RH for 2 and 4 wt % solutions (Figure 3c). At low RH, the upper-limit drying temperature minimally increased, although the corresponding films had numerous wrinkles. At high RH, flat films without wrinkles were obtained.

**Figure 3:**
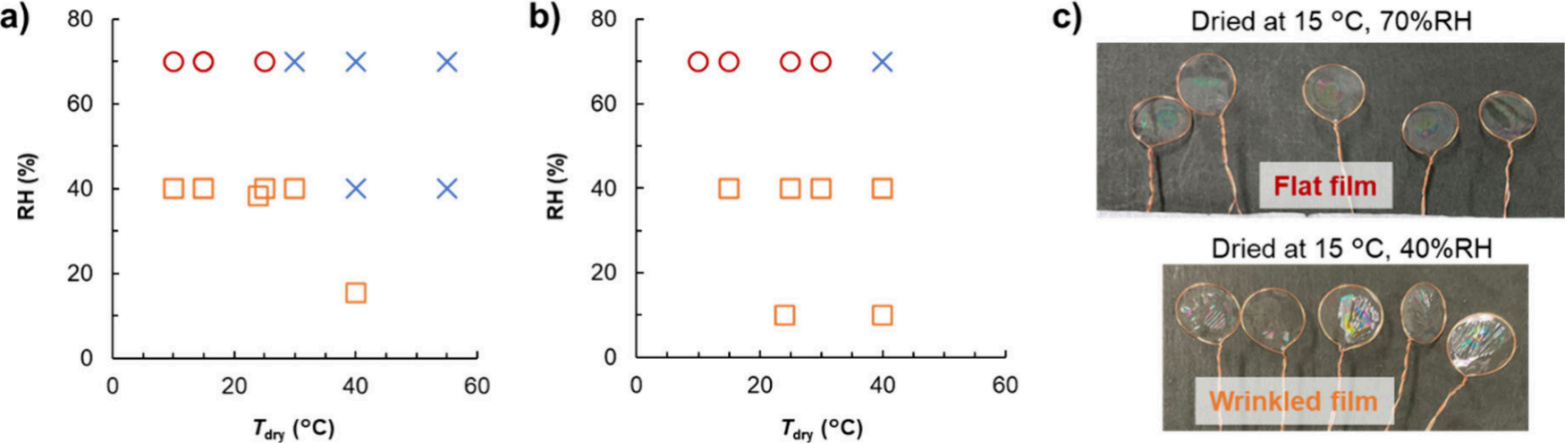
Effects of temperature and relative humidity (RH) on the formation of dried foam films from (a) 2 and (b) 4 wt % gelatin solutions. Film formation was tested five times using 1 cm–diameter frames to demonstrate good reproducibility. Symbols: dried films with flat surfaces (red circles) and wrinkled surfaces (orange squares), failure of film formation (blue crosses). (c) Photographs of dried gelatin films prepared using 2 wt % solution and 1 cm–diameter frames. Flat (upper) and wrinkled (lower) films were obtained by drying at 15 °C and RHs of 70% and 40%, respectively.

To visually track the liquid-film drying process, a 3 cm–diameter frame was immersed into a 4 wt % gelatin solution at 45 °C and then removed. The drying process was captured on video under atmospheric conditions (25 °C, 46%; ). Approximately 5 min after the recording had started, small wrinkles appeared on the inside of the frame, spreading throughout the film over the next 5 min. As the frame size increased, the upper-limit temperature at which dried films could be formed decreased, and wrinkles occurred more frequently. To illustrate the occurrence of wrinkles, one can imagine a flat elastic sheet with a free boundary pulled at two points on the circumference, with wrinkles forming in the direction perpendicular to the tensile stress (). We speculate that the same phenomenon occurred during the gelation of the thin liquid films through the following mechanism. When the liquid film gels, volume contraction creates tensile stress in the in-plane direction. As shown in , gelation does not occur simultaneously throughout the entire film, i.e., a time lag is observed between the areas that gel first and those that gel later. When gelation progresses around already gelated areas, the spatial variations in the in-plain tensile stress cause the stretching of these areas in the direction of strong tensile stress. When this stress field becomes sufficiently large, wrinkles appear in the direction perpendicular to the tensile stress gradient. When drying is performed at high RH, the volume contraction of the film during drying decreases, and so does the in-plane tensile stress. The facile movement of polymer chains under high-RH conditions allows tensile stress to be rapidly relaxed through changes in the chain conformation and the rearrangement of the gelled polymer network. Both of these effects inhibit wrinkle formation. Large liquid films are more prone to wrinkling because of the unbalanced in-plane tensile stress resulting from heterogeneous volume contraction during drying. In summary, the solution concentration and drying temperature strongly influence the feasibility of dry-film formation, and the RH during drying largely determines film flatness.

To understand the relationship between the film-forming capability and other properties of gelatin solutions, we measured their surface tensions and viscosities (Figures 4a and 4b). Increasing the solution concentration from 1 wt % to 5 wt % reduced surface tension by ∼ 5 mN/m. This reduction had a favorable effect on the formation of liquid films, as inferred from the mechanism of soap film formation. When the temperature was lowered from 60 to 30 °C, the surface tension increased by ∼ 5 mN/m, which disfavored the formation of thin liquid films. The surface tension was negatively correlated with the solution concentration and temperature, which was largely ascribed to the polymer chain structure.^43,44^ However, the surface tension change due to changes in temperature and concentration was small and showed an inverse trend, which suggests that surface tension was not a major factor influencing the formation of dried foam films. Conversely, the viscosity of gelatin solutions was strongly influenced by their concentration and temperature. Increasing the solution concentration from 1 wt % to 5 wt % increased the viscosity ∼ 100-fold. Cooling a 2 wt % solution from 60 to 30 °C increased the viscosity 60-fold. In particular, a substantial viscosity increase was observed as the temperature decreased to near-gelation-temperature values, possibly because of the formation of isolated clusters near the sol–gel transition.^45,46^ Thus, the solution viscosity substantially increased at high concentrations and low temperatures, i.e., under the conditions favoring dried film formation, which indicates a close relationship between the solution viscosity and film-forming ability.

**Figure 4:**
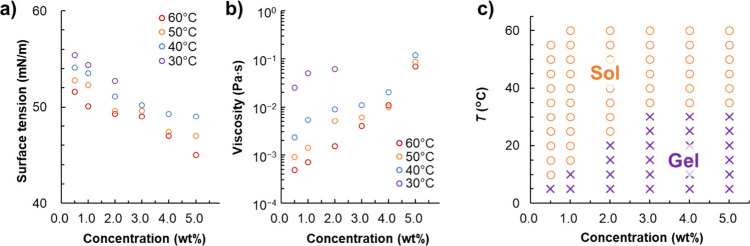
Effects of the gelatin solution concentration on (a) surface tension and (b) viscosity. (c) Sol–gel diagram illustrating the effects of gelatin solution concentration and temperature on the solution state (sol vs gel).

Gelatin dissolves in water at high temperatures and becomes a transparent gel at low temperatures.^47^ This thermoreversible gelation behavior was investigated using the test tube tilt method at solution concentrations of 0.5–5.0 wt % (Figure 4c). The sol state was stable at low concentrations and high temperatures, whereas the gel state was stable at high concentrations and low temperatures. The upper-limit temperature for gelation increased with the increasing solution concentration, in line with previous reports.^47,48^ The sol–gel transition temperature was compared with the upper-limit temperature at which dried foam films could be formed. At solution concentrations of 2 and 4 wt %, the upper-limit temperature for dried film formation was 10 °C higher than the corresponding sol–gel temperature. The close relationship between the sol–gel temperature and upper-limit dry-film-formation temperature suggests that gelation had a notable effect on the drying of thin liquid films.

To estimate the temperature change of the liquid film during drying, a 2 cm–diameter frame was immersed into a 4 wt % solution at 45 °C, removed, and kept in air at 24 °C and 43% RH. The approximate temperature of the liquid film was measured using an infrared thermometer 10–20 s after the onset of air exposure (). The temperature of the liquid film immediately after removal from the solution exceeded the room temperature but fell below it after ∼ 50 s and returned to room temperature after ∼ 90 s, subsequently remaining constant.

By interpolating the data between 0 and 5 min in , we estimated the weight change rate of the liquid film as 10%/min, which indicated that water evaporation was moderate in this time range. Therefore, the temperature change may be caused by cooling due to water evaporation from the liquid film as well as by forced cooling by convection due to room-temperature air in contact with the surfaces of the liquid film. According to the video of the drying process of a thin liquid film (), wrinkles formed between 8–10 min, i.e., the liquid film had solidified by this point at the latest. The average gelatin concentration of the liquid film was estimated from its weight change during drying at room temperature (). The gelatin concentration 8–10 min after the onset of drying was 5–25 wt %, suggesting that a sufficient amount of liquid remained in the gelled film.

Andrieux et al. investigated the drying process of a calcium ion–cross-linked alginate gel using a home-built microfluidic thin-film pressure balance apparatus. When the holding time before drying was prolonged and approached the gelation time, an intact dry film could be formed.^24^ In the study of Chen et al., a thin liquid film prepared from an aqueous dispersion of graphene oxide nanosheets was dried using a lyotropic liquid crystalline phase to obtain a centimeter-sized freestanding dried foam film with multilayered graphene nanosheets.^23^ These results, as well as those presented herein, suggest that the intermediate states of viscoelastic solids containing water, such as gels and lyotropic liquid crystals, are suitable for the drying of thin liquid films to obtain large-area freestanding dried foam films.

Based on these results and the film morphology diagram presented by Andrieux et al., we proposed the following formation mechanism of dried gelatin foam films as schematically illustrated in Figure 5. Initially, the frame is immersed into a gelatin solution and then removed, which causes the liquid film to adhere to the frame. Low surface tension and high viscosity of the solution transiently maintain the liquid film on the frame. When the liquid film comes into contact with air and some of the water evaporates, its temperature drops, and gelatin solidifies into a gelatinous thin film because of thermoreversible gelation. This gelatinous film exhibits good mechanical strength and thus withstands disturbances without rupturing in the subsequent drying process. During drying, the gradual evaporation of water from the gelled film reduces its thickness and increases its density, resulting in a uniform, intact, dry, and dense freestanding thin film. When the solution concentration is low, the frame is large, and the drying temperature is high, the liquid film has a short lifespan and gelation is slow, rupturing before gelation occurs. When the solution concentration is high, the frame is small, and the drying temperature is low, the liquid film has a long lifespan and gelation is fast, i.e., gelation progresses before the liquid film ruptures. When the entire thin film gels, it remains stable and becomes thinner and denser as water evaporates, which results in a mechanically stable, homogeneous, and dry freestanding thin film.

**Figure 5:**
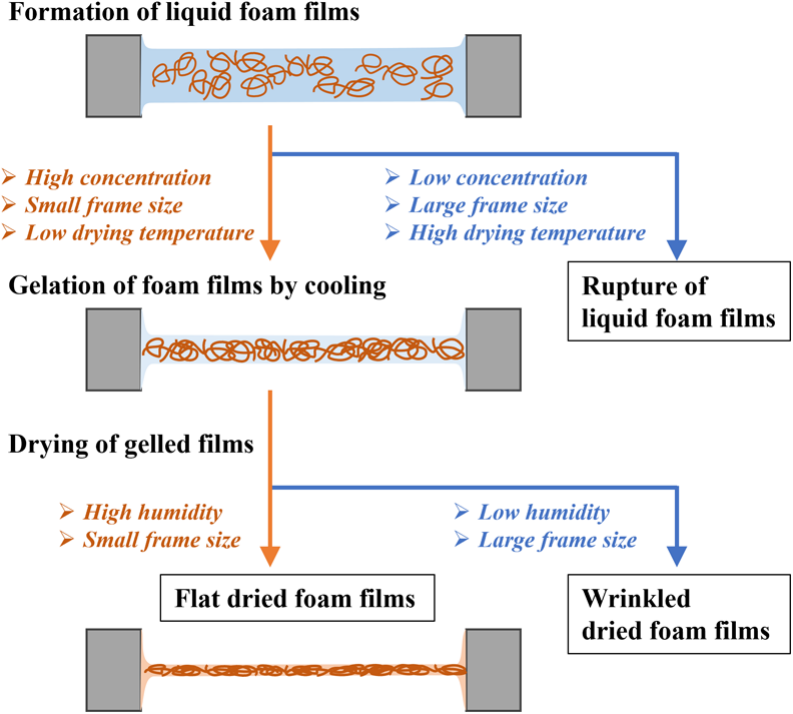
Schematic of the process responsible for the gelling of liquid foam films and formation of large-area, flat, and dried foam films under controlled drying conditions.

Benattar et al.^49^ developed a procedure for transferring thin films to solid substrates. When a thin film is formed on a solid substrate, its direct transfer to another substrate is typically difficult and often results in cracking, pinhole formation, and/or fracture due to insufficient mechanical strength and/or brittleness. As our gelatin films were free-standing, they could be easily transferred to other substrates, such as a porous polycarbonate membrane or silicone rubber, via simple attachment to the same. The films were stable under vacuum and could be selectively surface-functionalized by thermal deposition or sputtering methods in vacuum. A thin platinum layer was coated on the surface using an argon-ion sputterer, and a carbon thin film was deposited without deteriorating the film (). Furthermore, the films were mechanically stable under reduced pressures of up to 90 kPa and could therefore be possibly used as a separation membrane for the recovery and purification of gases and vapors.

Several reagents can be used to cross-link gelatin films in aqueous and organic media,^50,51^ as exemplified by formaldehyde, glutaraldehyde, carbodiimide, dextran dialdehyde, and genipin. Herein, dried gelatin films were cross-linked using four cross-linking agents at different concentrations and reaction times (). Gelatin films cross-linked with glutaraldehyde showed no noticeable changes after exposure to hot water (60 °C) for 3 days and even after 10 days, which highlights the efficiency of glutaraldehyde as a cross-linking agent for dried gelatin films. In addition, the incorporation of free aldehyde groups upon cross-linking with glutaraldehyde can enhance the bioadhesiveness of gelatin films, with the extent of this enhancement increasing with the content of these groups.^52^

The cross-linking of the hydrophilic gelatin films makes them insoluble in water and therefore suitable for the removal of nanocolloid particles and dye molecules from aqueous media. Herein, we cross-linked gelatin films using glutaraldehyde and tested the water purification performances of the resulting membranes. The thicknesses of the films prepared from 5 and 3 wt % gelatin solutions were determined by SEM as 2.5 and 1.5 μm, respectively. The results of permeability and rejection performance testing are summarized in Table 1. A low glutaraldehyde concentration of 1% resulted in a high water flux and poor rejection performance (63% for 5 nm gold colloids). When the glutaraldehyde concentration was increased to 5% or 10%, the water permeability decreased, and the rejection performance improved. In particular, the film cross-linked with a 10% glutaraldehyde solution achieved a 100% rejection for 5 and 2 nm gold colloids, Direct Yellow 1 (), and potassium ferricyanide. These results demonstrate the potential of cross-linked gelatin films as water separation membranes.

**Table 1:** Water Purification Performances of Dried Foam Films Crosslinked with Glutaraldehyde^a^

Gelatin thin film membrane		GTF1	GTF2	GTF3	GTF4	GTF5
Gelatin concentration (%)		5	5	5	5	3
Cross-linker concentration (%)		1	5	10	10	10
Cross-linking sides		Both	Both	Both	One	One
Filtrate solution (in water)	Parameter (unit)					
Pure water	Flux (L/(m^2^ h))	26.0	20.0	4.5	5.0	11.0
5 nm Au	Flux (L/(m^2^ h))	22.5	16.6	3.3	3.4	10.5
	Separation (%)	63	88	100	100	91
2 nm Au	Flux (L/(m^2^ h))	25.0	18.3	3.4	3.6	N.D.^a^
	Separation (%)	20	30	100	100.0	–
Direct Yellow 1	Flux (L/(m^2^ h))	N.D.^a^	N.D.^a^	5.0	3.8	N.D.^a^
	Separation (%)	–	–	100	100	–
K_3_[Fe(CN)_6_]	Flux (L/(m^2^ h))	N.D.^a^	N.D.^a^	3.1	3.7	10.0
	Separation (%)	–	–	100	100	40

a The gelatin thin films were prepared by drying at room temperature using 2.5 cm-diameter frames.

b N.D.: Separation experiments were not performed.

Gelatin is highly soluble in water and has surface-active properties similar to those of surfactants, which are suitable for the formation of dried foam films. Although other surface-active proteins, e.g., albumin, gliadin, and lactalbumin are known, they are less soluble in water than gelatin and cannot stabilize dried foam films on their own because of their low concentrations. In contrast, when glycerol was added to an aqueous albumin solution, dried albumin foam films were obtained (). Glycerol increased the viscosity of the albumin solution and possibly acted as a plasticizer in the corresponding film. By dissolving a surface-active protein in water and making the solution viscosity sufficiently high, one can obtain dry, thin, large-area, mechanically stable, and free-standing films.

In the future, biocompatible protein thin films, by possessing functional groups, hold the potential to realize novel bioapplications that combine mechanical strength with selective permeability to gases and liquids. Thinner dried gelatin films with sufficient mechanical strengths should be fabricated to improve the separation performance of the corresponding membranes. Films exhibiting elasticity in the dry state are expected to be strong and rupture-resistant. Enhancing the molecular relaxation of polymer chains would help form large-area, wrinkle-free, homogeneous thin films. Dried foam films containing nanoparticles and nanofibers are expected to provide new functionality to thin foam films, which may be prepared using gelatin solutions containing these nanofillers. One should also verify whether the mechanism of dried foam film formation proposed herein is applicable to other synthetic polymers and macromolecular surfactants.

## Conclusion

Ultrathin, freestanding, and easily transferable centimeter-sized gelatin thin films were produced by air-drying aqueous gelatin solutions with optimal properties under optimal conditions. When a gelatin solution with a concentration of ≥ 1.5 wt % was applied to a polymer mesh with an aperture of ≤ 5 mm, a coverage of almost 100% was obtained for a 10 cm × 10 cm area. The thickness of the dried foam film decreased with the decreasing gelatin concentration, which indicates a trade-off between low film thickness and high coverage. Dried foam films could be formed reproducibly at drying temperatures of ≤ 25 and ≤ 30 °C for 2 and 4 wt % solutions, respectively, using a 1 cm–diameter frame. The upper-limit drying temperature was 10 °C higher than the gelation temperature, suggesting that the gelation behavior of gelatin dominated the formation of dried foam films. While low surface tension promotes the formation of liquid films, high solution viscosity prolongs the lifetime of the liquid film, enabling gelation. At a high solution concentration, small frame size, and low drying temperature, gelation proceeded before the liquid film ruptured. Once the entire thin film was gelatinized, it remained stable, and as water evaporated, the thin film became thinner and denser. Hence, a stable, homogeneous, freestanding, dried foam film with an excellent mechanical strength was obtained. Low-RH drying afforded films with numerous wrinkles, whereas high-RH drying resulted in flat wrinkle-free films. This wrinkling was probably caused by an imbalance in in-plane tensile stress due to heterogeneous volume contraction during drying. The dried gelatin films were stable under vacuum and could be selectively surface-functionalized by thermal evaporation or sputtering in vacuum. Films cross-linked with glutaraldehyde were stable in hot water and achieved 100% rejection for 2 and 5 nm gold colloids, Direct Yellow, and potassium ferricyanide. These results highlight the potential of cross-linked gelatin films as water separation membranes.

## Supplementary Material





## Notes

Additional scanning electron microscopy micrographs, weight and temperature changes during drying, photographs during drying, and digital photographs of surface-coated dried foam films, and summaries of gelatin film-lifting, results of the hot-water stability test, and albumin film-forming conditions (PDF)
Movie S1: movie of drying process under atmospheric conditions (25 °C, 46%) (MP4)

## ABBREVIATIONS

FTIR: Fourier transform infrared
SEM: scanning electron microscopy
RH: relative humidity
