# Supplementary material for: Fabrication of Free-Standing Gelatin Thin Films via the Gelation and Drying of Liquid Foam Films
Source: Langmuir. 2025 Oct 14;41(42):28637–45. doi: 10.1021/acs.langmuir.5c03893 (PMC12573788; doi:10.1021/acs.langmuir.5c03893)
Supplement: Supplementary file 1 [file la5c03893_si_001.pdf]

Supporting Information

Fabrication of Free-Standing Gelatin Thin Films via the Gelation and Drying of Liquid Foam Films

Ashesh Garai,[#] Sadaki Samitsu,[^]* Miwa Ohniwa,[^] Izumi Ichinose[^]

[#]Rammohan College, 102/1 Raja Rammohan Sarani, Kolkata 700009, India

[^]Research Center for Macromolecules and Biomaterials, National Institute for Materials Science, 1-2-1 Sengen, Tsukuba, Ibaraki 305-0047, Japan

[*]To whom correspondence should be addressed.

E-mail: SAMITSU.Sadaki@nims.go.jp

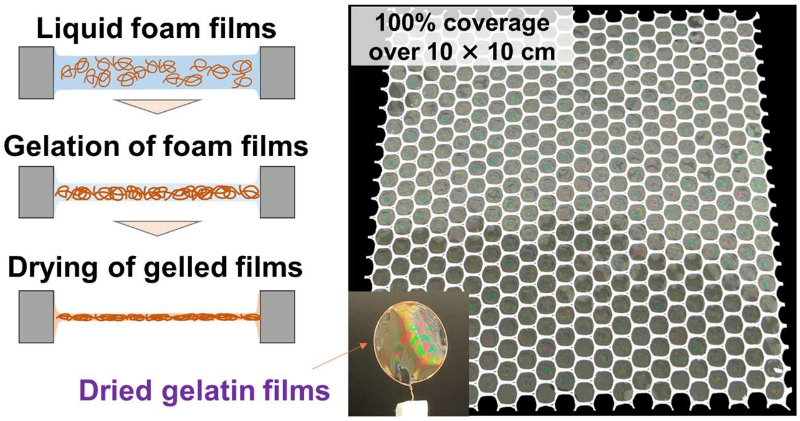

Number of pages: 7

Number of figures: 6

Number of tables: 3

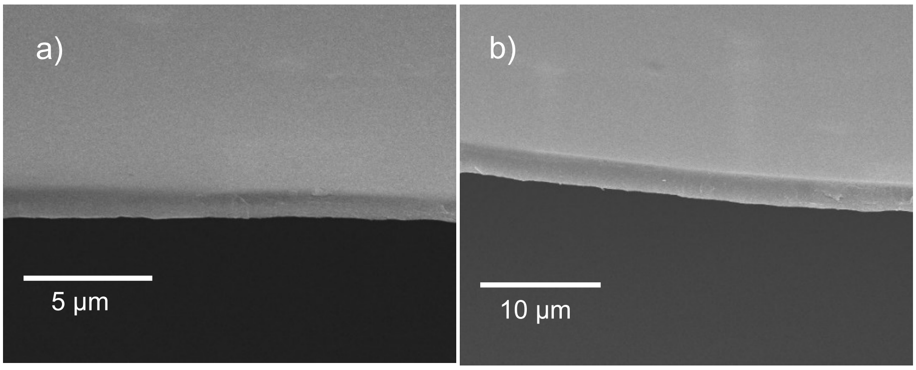

Figure S1. Scanning electron microscopy micrographs of gelatin foam films prepared from (a) 3 and (b) 5 wt% solutions on a 2 cm-diameter copper frame.

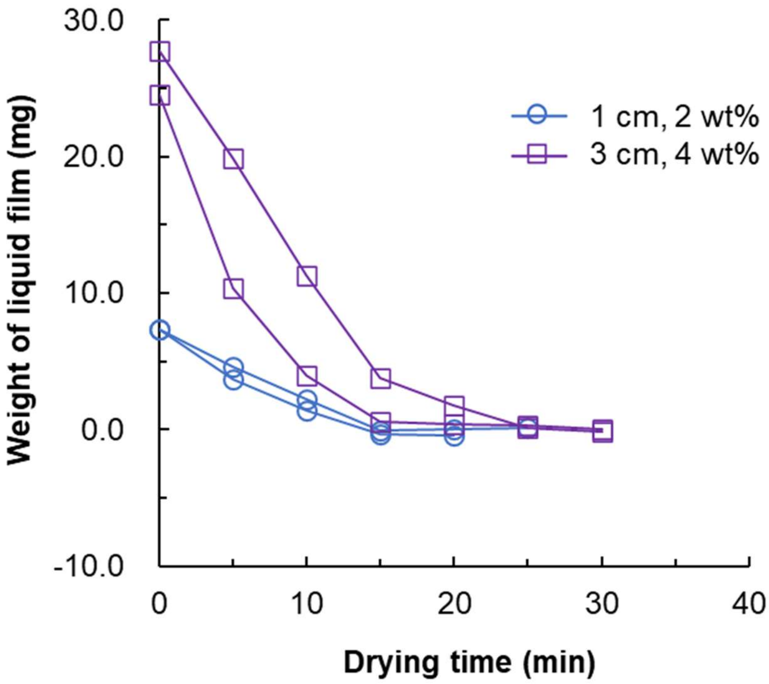

Figure S2. Weight change of liquid foam films over time left under atmospheric conditions (24 ± 2 °C, humidity 35–45%).

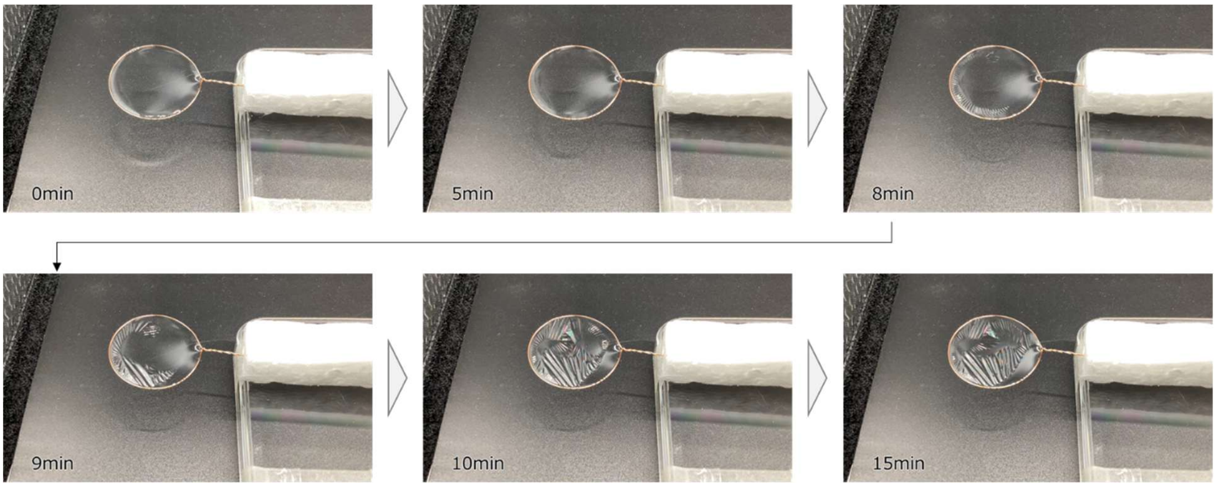

Figure S3. Photographs of drying process of a liquid foam film left to dry over time under atmospheric conditions (24 °C, humidity 46%). The film was prepared from 4 wt% solution using a 3 cm-frame. See Movie S1.mpg for details.

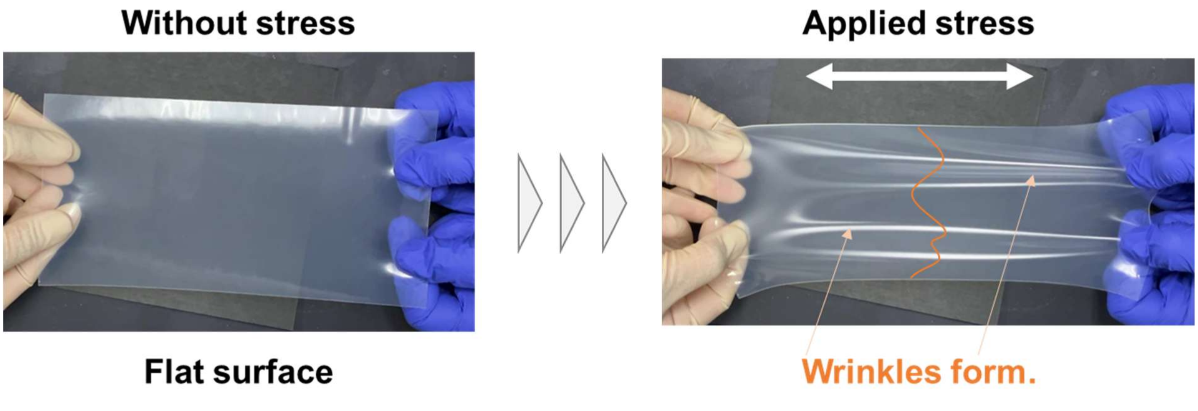

Figure S4. Demonstration of wrinkle formation in a silicone rubber sheet when tensile stress is applied.

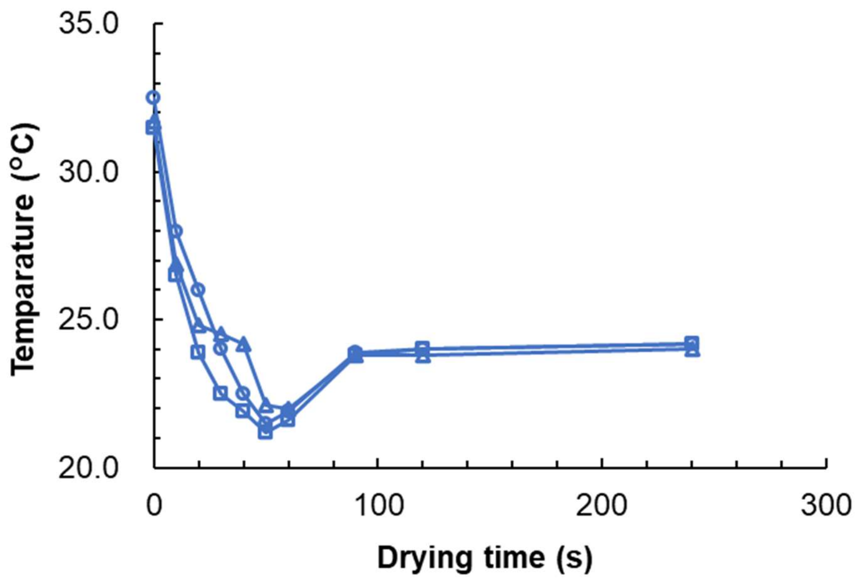

Figure S5. Approximate temperature of the liquid foam film was measured using an infrared thermometer. A 2 cm-diameter frame was immersed in a 4 wt% solution at 45 °C, removed from the liquid, and kept in air at 24 °C and 43% relative humidity. The experiment was repeated three times and the results plotted with different symbols.

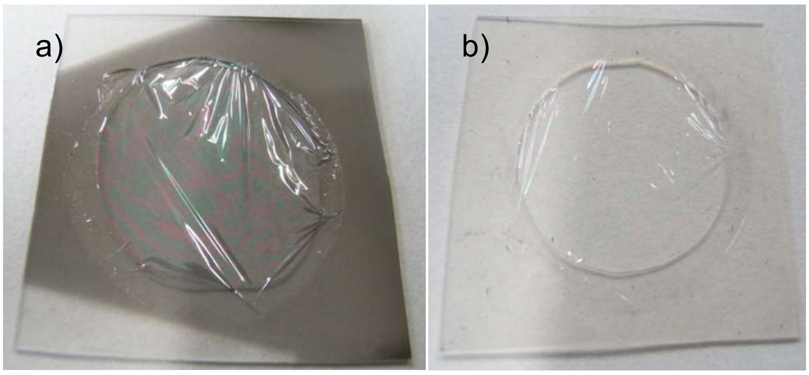

Figure S6. Photographs of dried foam films prepared from 3 wt% gelatin solution and coated with (a) platinum and (b) carbon.

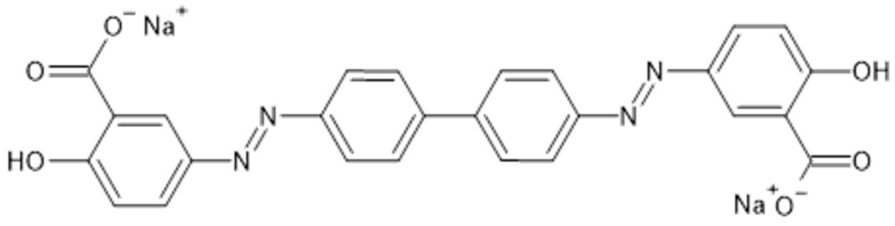

Figure S7. Chemical structure of Direct Yellow 1.

Table S1. Effects of lifting speed and copper frame position on gelatin film formation (1 wt% gelatin solution, 55 °C).

Position of copper frame	Lifting speed (mm/s)	Conclusion
Vertical (90°)	0.1–0.3	No film
	0.3–10	No film
	10–30	No film
Horizontal (0°)	0.1–0.3	No film
	0.3–10	Stable film
	10–30	Stable film
Inclined (45°)	0.1–0.3	No film
	0.3–10	Stable film
	10–30	Stable film with occasional rupture

Table S2. Summary of stability of crosslinked gelatin films in hot water. Crosslinking treatments were tested for four crosslinking agents at different concentrations and reaction times.

ID	Crosslinking agents	Medium	Concentration (wt%)	Time (h)	Stability in hot water (60 °C)
GA1	Glutaraldehyde (50 wt% aqueous solution)	water/ethanol mixture	0.5	3	Stable for 1 day
GA2			2.0	2	Stable for 1 day
GA3			5.0	1	Stable for 1 day
FA1	Formaldehyde (37 wt% aqueous solution)	water/ethanol mixture	1.0	3	Soluble
FA2			1.0	9	Soluble
FA3			1.0	24	Stable for 1 day
DIC1	*N*,*N*'-Dicyclohexylcarbodiimide	xylene	0.5	3	Soluble
DIC2			0.5	24	Soluble
DIC3			5.0	3	Soluble
DIC4			5.0	24	Soluble
DIC5			10.0	1 week	Soluble
PF1	Paraformaldehyde	gas phase (70 °C)	100 mg in 100 mL bottle	2	Soluble
PF2				24	Stable for 1 day

Table S3. Summary of albumin film formation conditions.

Sample	Albumin (wt%)	Glycerol (mL)	Temperature (°C)	Film characteristic	Thickness (μm)
Set-1	1	0	30	Film immediately ruptured	-
Set-2	1	0	55	Film immediately ruptured	-
Set-3	3	0	55	Film immediately ruptured	
Set-4	5	0	55	Film immediately ruptured	-
Set-5	5	3	55	Stable film formed	2.5 ± 0.5
Set-6	5	2	55	Stable film formed	2.3 ± 0.5
Set-7	3	2	55	Stable film formed	1.3 ± 0.4
Set-8	1	2	55	Stable film formed	0.8 ± 0.3
Set-9	1	1	55	Film immediately ruptured	-
Set-10	1	1.5	55	Film immediately ruptured	-
